# Identification of C-Kit-Positive Interstitial Cells in the Dog Lower Urinary Tract and Relationship with Smooth Muscle and Nerves. Hypotheses for a Likely Pacemaker Role.

**DOI:** 10.4061/2010/981693

**Published:** 2010-07-25

**Authors:** Silvana Arrighi, Giampaolo Bosi, Debora Groppetti, Fausto Cremonesi

**Affiliations:** ^1^Laboratorio di Anatomia, Dipartimento di Scienze e Tecnologie Veterinarie per la Sicurezza Alimentare, Università degli Studi di Milano, Via Trentacoste 2, 20134 Milano, Italy; ^2^Dipartimento di Scienze Cliniche Veterinarie, Sezione di Clinica Ostetrica Veterinaria, Università degli Studi di Milano, Via Celoria 10, 20133 Milano, Italy

## Abstract

The aim of this work was to give an evidence of the likely presence of interstitial cells in the canine lower urinary tract and to study their possible interactions with the musculature and the intramural innervation. Cryosections of normal canine bladder and urethra were immunofluorescently labelled with c-kit, a transmembrane, tyrosine kinase growth factor receptor, known to be expressed on the interstitial cells of Cajal (ICCs) of the gut. The relationship with antiactin positive smooth muscle cells and PGP9.5-positive intramural innervation was also investigated by confocal microscopy. Anti-c-kit labelling demonstrated a network of elongated and branched c-kit positive cells, which were located in interstitial spaces, oriented in parallel to the smooth muscle bundles that form the bladder muscular layer, irrespective of dog sex. Cells with a similar localization were also PAS- and NADPH-diaphorase-positive. A contact between c-kit immunofluorescent cells and intramural innervation was demonstrated, too. The roles of interstitial cells might include regulation of smooth muscle activity of the bladder detrusor, integrating neuronal signals during urine storage and voiding.

## 1. Introduction

The two main and correlate functions of the organs belonging to the lower urinary tract are the storage and periodic elimination of urine, which are basically mediated by contraction of the muscular layer and are regulated by the somatic, sympathetic, and parasympathetic innervations, synergically working. Higher order modulation is from the nervous centers of the cerebral cortex, the cerebellum, and pons. In recent years, many investigators have suggested that a network of cells triggering spontaneous contractions and myogenic slow wave activity of the muscular wall in the urinary tract of many mammalian species may exist [1–5]. This is a current and topical research area in functional urology, with possible interesting rebounds on different pathologies [6].

In the intestinal muscular layer, the presence of a harmonized activity necessary for movement is well known and is operated, in the human [7] as well as in animal species [8, 9] including the dog [10], by the intricate network of the so-called interstitial cells of Cajal (ICCs). In analogy with this system, many authors have invoked an ICC-like system in the urinary tract, coordinating the urinary “peristalsis” [4]. A similar network of cells has been described as strategically located beneath the urothelium in guinea-pigs, and humans [11–14], or at the level of the muscular layer as well, in rabbits, guinea-pigs and human [4, 15–17]. Some authors described the presence of several populations of ICC-like cells, either located below the urothelium, in the lamina propria region, and throughout the detrusor [18, 19], making connections with intramural nerves and closely associated with smooth muscle cells, in the detrusor. No reports exist of the presence of these cells in the lower urinary tract of the dog. Nevertheless, it is possible to conceive that abnormalities of such an intrinsic system of motility control in the dog urinary tract might be at the basis of a variety of important urological diseases, such as the urethral incompetence mechanism, an acquired condition thought to be a consequence of a dysfunction in the musculature innervation [20]. Unequivocal methods usually used for the demonstration of ICC are electron microscopy and a growing number of markers applicable to bright field and fluorescence microscopy [10]. C-kit is a transmembrane, tyrosine kinase growth factor receptor expressed on fetal and adult cells, including the interstitial cells of Cajal of the gut. Also useful, although not definitive as sole tests, are the histochemical reactions for diaphorases [10], and Periodic acid-Shiff (PAS) reactivity due to the high glycogen content in ICC [21]. 

Following our interest in the study of innervation of the lower urinary tract in the dog [22], this work is aimed at demonstrating the likely presence of interstitial cells (ICs) in the canine lower urinary tract. Over-mentioned tools were utilized for this purpose, together with the general neuronal marker protein gene product (PGP) 9.5, which was previously employed with the aim to mark nerve fibres in relation to IC cells [14].

## 2. Materials and Methods

### 2.1. Animals

Samples were collected at the Department of Veterinary Clinical Science of the Faculty of Veterinary Medicine of Milan from adult dogs of both sexes (3 males and 3 females), which were euthanized after diagnoses excluding urinary pathologies (mainly trauma). Organ sampling was done after acceptance of the dog owners. It was verified that the organs of the lower urinary tract were healthy. Several fragments were obtained within 10 minutes of euthanasia from the bladder apex, body, and neck, and from the urethra, proximal to the bladder neck. The male urethra was collected at the level of preprostatic part. Fragments of healthy small intestine were collected as well, to be used as positive controls.

### 2.2. Tissue Processing

Tissue fragments were immersed in 4% paraformaldehyde in 0.01 M phosphate-buffered saline (PBS) pH 7.4 for 24 h fixation at 4°C. Samples were either (i) dehydrated in a graded series of ethanol, cleared in xylene and paraffin embedded, or (ii) cryoprotected overnight by infiltration with a 20% sucrose solution in PBS at 4°C, then snap frozen in liquid nitrogen-cooled isopenthane, using OCT (Tissue-Tek, BDH, UK) as an embedding medium. Either deparaffinized microtome sections (4–6 *μ*m thick) or cryosections (10–20 *μ*m thick) were stained with routine stains, haematoxylin/eosin, Azan, or Mallory trichromic stain, for morphological purposes. Successive microtome or cryo-sections of the specimens were processed as follows.

### 2.3. Histochemistry

Periodic Acid-Shiff (PAS) reaction, which is known to selectively stain neutral glycoconjugates, was performed on deparaffinized sections. Conventional PAS reaction, with and without previous diastase digestion, was performed with the aim to demonstrate the presence of glycogen. 

Cryostat sections picked up on gelatin-coated glass slides were histochemically treated for demonstration of NADPH-diaphorase or NADH-diaphorase according to Xue et al. [10]. Both histochemical reactions were described as possible markers of the intestinal ICC [21]. Sections were incubated in PBS pH 7.4 containing 1 mM *β*-NADH or 1 mM *β*-NADPH (Sigma, Italy) and 0.6 mM nitroblue tetrazolium (Sigma) for 10–30 min at 37°C [10]. After incubation with either the solutions for NADPH-d or NADH-d the sections were rinsed in PBS, exsiccated and mounted in Eukitt (Bioptica, Milan, Italy). The specificity of the stains was verified by excluding NADH and NADPH, respectively, from the incubating media, which in both cases abolished all the activities.

### 2.4. Immunohistochemistry and Double Immunofluohistochemistry

On microtome sections, standard immunohistochemical techniques were employed to test each primary antiserum, according to previously described methods [22], which are briefly summarized. After dewaxing, 4 *μ*m-thick sections were washed and immersed in a freshly prepared 3% H_2_O_2_ solution for 15 min to block the endogenous peroxidase activity, followed by incubation in 1 : 20 normal goat serum (code X0907, DakoCytomation, Denmark) in Tris-Buffered Saline (TBS: 0.05 M Tris/HCl, 0.15 M NaCl) for 30 min to prevent background prior to incubation with primary antiserum. Sections were then incubated overnight in a humidity chamber at room temperature using the antibodies listed in Table 1, at the respective dilutions. Peroxidase-antiperoxidase complex (PAP, DakoCytomation) was employed to develop the reaction, thereafter the immunoreactive sites were visualized using a freshly prepared 3,3′-diaminobenzidine tetrahydrochloride (DAB, Sigma, Italy) solution. Sections were counterstained with Mayers' haematoxylin, dehydrated, and mounted using Eukitt (Bioptica, Milan, Italy). The specificity of immunostaining was verified (1) by omission of the 1st layer; (2) by the use of nonimmune mouse or rabbit serum in place of the primary antiserum at the same dilution. The results of these controls were negative. Positive controls were performed utilizing sections of dog gut. 

Histochemical and immunohistochemical stainings were evaluated and photographed under an Olympus BX50 photomicroscope, equipped with a digital camera and DP software (Olympus, Tokyo, Japan) for computer-assisted image acquirement and managing. 

Immunofluorescence histochemistry was conducted on 20–40 *μ*m thick cryostatic sections. After inhibition of non-specific reactivity with 1 : 20 Normal Goat Serum (DakoCytomation) in Tris-HCl-buffered saline (TBS; 0.05 M, pH 7.4, 0.55 M NaCl) for 30 min, the sections were incubated with the first primary antiserum (anti c-kit) overnight at 4°C. Slides were washed in TBS with 0.1% Triton-X 100 (TBS-T), and then treated with the biotin-avidin blocking kit solutions (Vector Laboratories, Burlingame, CA, USA). After being rinsed in TBS-T, the sections were incubated with 10 *μ*g/mL goat biotinylated antimouse IgG (Vector Labs.) in TBS-T for 1 h at RT, thereafter, were treated with 10 *μ*g/mL Fluorescein Avidin D (Vector Labs.) in 0.1 M NaHCO_3_ pH 8.5 with 0.15 M NaCl for 1 h at RT. Double label of sections was obtained by the sequential addition of a second primary antibody, either antiactin or anti-PGP9.5, thought to be a general nerve marker [23], or anti-NOS1, followed by the suitable secondary antibody (see Table 1) for 1 hour at RT, and labelled with 10 *μ*g/mL Rhodamine Avidin D (Vector Labs.) in 0.1 M NaHCO_3_ pH 8.5 with 0.15 M NaCl for 1 hour at RT. Staining with fluorescein-avidin DCS (Vector Labs.) was also performed to exclude mast cell staining by c-kit [5]. Finally, stained sections were embedded into Vectashield Mounting Medium (Vector Labs.) and observed under a confocal laser scanning microscope Olympus Fluoview FV300 (Olympus, Tokyo, Japan), using excitation wavelengths of 488 and 668 nm, from a krypton-argon laser and a green Helium-Neon laser and barrier filters set for flurescein isothiocyanate and rhodamine. Images of superimposed fluorescence were obtained after acquiring the image slice of each laser channel sequentially. 

## 3. Results

**Figure 1:**
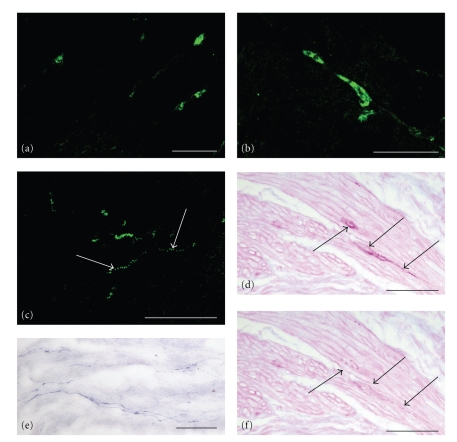
(a) Dog jejunum, anti-c-kit immunofluorescence. Interstitial cell bodies can be seen distributed in the smooth muscle wall. (b) Dog bladder, anti-c-kit immunofluorescence. A compressed image obtained by confocal microscopy in a thick tissue section shows strong immunoreactivity in the cytoplasm of an interstitial cell, which is distended among a group of smooth muscle cells. (c) Dog bladder, anti-c-kit immunofluorescence, image obtained as in (b). Subtle c-kit-IF interstitial cells can be seen, elongated among smooth muscle cells and branching with long processes, discernible as immunofluorescent bright dots (arrows). (d) Dog bladder, PAS reaction. Reactivity is present in the cytoplasm of few interstitial cells (arrows), elongated among the smooth muscle bundles. Note the similarity of aspect and localization compared to the c-kit-IF cells shown in (b). When transversally cut, ICs show a roundish shape. Nuclei are unstained. (e) Dog bladder, NADPH-d reaction. Reactivity is present in interstitial cells, running parallel to each other, whose aspect and localization are very similar to the c-kit-IF cells shown in (c). (f) Dog bladder, PAS reaction after diastase digestion. PAS-negativity in a serial section confirms the large accumulation of glycogen granules in the cytoplasm of ICC-like cells shown in (d). Scale bars = 25 *μ*m.

Identification of c-kit-immunoreactive cells was extremely difficult to obtain in samples of the lower urinary tract of dogs. Several attempts were firstly made by following the antibody producer's suggestion, using paraffin-embedded tissues with the recommended working dilution, incubation time and temperature, as well as antigen unmasking with high temperature. Only by immunofluorescence techniques on thicker frozen sections, employ of double antibody concentration (1 : 10 instead of 1 : 20–1 : 40, recommended by manufacturer), and observation under a confocal microscope, it was possible to recognize consistent c-kit-labelling in tissues from at least five dogs. Actually, c-kit-immunofluorescent (IF) cells, recognizable in the gut wall (Figure 1(a)), were few in the dog lower urinary tract, and localized only in the bladder wall (Figure 1(b)). Immunopositive cells were elongated and showed slender processes, distributing through the depth of a thick section, discernible as immunofluorescent bright dots (Figure 1(c), arrows), whose path was possible to be followed only by confocal microscopy. C-kit-immunoreactive cells were mostly located in interstitial spaces on boundary of smooth muscle fascicles that form the bladder detrusor, longitudinally oriented, in parallel with the smooth muscle cells. Interstitial cells were stained by PAS reaction (Figure 1(d)), being clusters of cytoplasmic granules responsible of the PAS positivity. PAS negativity after diastase digestion confirmed the large accumulation of glycogen granules in the cytoplasm of these cells (Figure 1(f)). Interstitial cells stained positively by NAPDH-diaphorase technique (Figure 1(e)). In thick sections of frozen formaldehyde-fixed samples, ICs were arranged to form a three-dimensional network, contacting each other above and below the plane of focus through their long processes. Slender NADPH-d reactive cells showed an aspect and localization very similar to the c-kit-IF interstitial cells (compare Figure 1(c) with Figure 1(e)).

**Figure 2:**
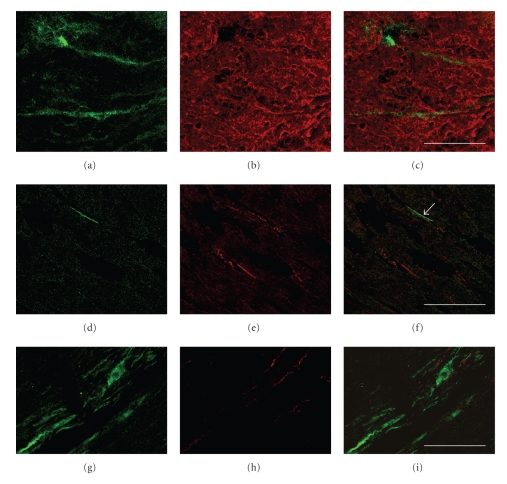
[(a), (b), (c)] Dog bladder, c-kit, and actin double immunofluorescence. Two green-labelled c-kit-IF cells can be seen in the bladder musculature (a). Anti-actin-IF smooth muscle bundles of the detrusor are rhodamine redstained (b). Superimposed image (c) demonstrates the localization of ICs in close association with the smooth muscle cells. [(d), (e), (f)] Bitch bladder, c-kit, and PGP9.5 double immunofluorescence. One green-labelled c-kit-IF cell (d) is present in the bladder muscular layer. Anti-PGP9.5-IF nerve fibres are rhodamine redstained (e). Overlay shows the close association of the IC and a PGP9,5-positive nerve fibre [(f), arrow]. [(g), (h), (i)] Bitch bladder, c-kit and NOS-I double immunofluorescence. Green-labelled c-kit-IF cells (g) are present in the bladder muscular layer. Anti-NOS-I-IF nerve fibres are rhodamine redstained (h). Overlay shows that ICs and nitrergic nerve fibres are closely associated (i). Scale bars = 25 *μ*m.

The localization of ICs on boundary of smooth muscle fascicles was confirmed by double labelling with anti-c-kit (Figure 2(a)) and antismooth muscle actin immunoreaction (Figure 2(b)). ICs were longitudinally oriented, in parallel with the smooth muscle cells (Figure 2(c)).

The relationship between bladder ICs and intramural innervation was investigated by double immunofluorescence experiments using c-kit antibody followed by the antibody protein PGP9.5, known to be a general phenotypic marker at the level of the peripheral nervous system. An association was occasionally noticed between c-kit-IF cells (Figure 2(d)) and PGP9.5-IF (Figure 2(e)) nerve fibres innervating the bladder wall (Figures 2(d), 2(e), and 2(f)).

NOS1-immunoreactive components of innervation were scarce either in bladder or in urethral wall. Rarely, a relationship of close vicinity of ICs to NOS-I-immunoreactive nerve fibres was also detected at bladder level (Figures 2(g), 2(h), and 2(i)).

## 4. Discussion

This paper is the first report demonstrating ICC-like cells in the canine urinary tract. The techniques utilized in our study for urinary ICs localization are recognized among the best markers for intestinal ICs in light microscopy [21]. Histochemical methods such as PAS and NADPH-diaphorase reactions were never utilized before in the identification of ICs in the urinary tract. Nevertheless, PAS and NADPH-diaphorase positivity confirmed to be a very useful tool in our specimens to identify ICs, a cell type that we demonstrated to be rare. Actually, in dog specimens, investigation on the ICs *in situ* was difficult, even in the intestinal wall which was used as positive control for immunoreactions, owing to their paucity, small dimensions, and sparse distribution in the smooth muscle layer of any organ wall. Moreover, use of immunofluorescence techniques and observation under confocal microscope was imperative to detect these slender and branching cells, utilizing thick tissue sections. Another initial difficulty in our research was linked to the fact that ICs appear to be present in the bladder wall only and not in the urethra. This finding is in contrast with the descriptions given in many species, such as the rodents, man [18], boar [24], and rabbit [2, 17]. In the male, pacemaker roles have been hypothesized at urethral level, acting in the regulation of smooth muscle activity including, beside control of bladder voiding, an intervening in seminal emission during ejaculation [24] and penile physiology [16]. Similar function was ascribed to ICs which were identified at vas deferens level [25]. 

No suburothelial ICs were detectable, in contrast with the descriptions given in literature for human [11, 12] and guinea pigs [14].

In a recent review, Brading [15] observed that the common function of the bladder in mammals is to store and expel urine, thus similarities in the properties of the detrusor in all species must exist. However, this author puts forward very interesting suggestions regarding functional differences in those mammals that use urine as a territorial scent marker, since this requires a mechanism to produce small spurts of urine in addition to a mechanism that will empty the bladder. This observation can also be at the basis of the presence of ICC-like cells in the bladder detrusor, perhaps responsible for the occurrence of phasic contractions. When present in the urethral wall and not in the bladder, as for instance in the boar [24], the function of ICs might be to prevent leakage of urine during filling by generating a urethral closure pressure, and to allow voiding at micturition. In this respect, the physical constraints might vary between the species.

Double immunofluorescence experiments on dog specimens have shown that c-kit-IF cells lie in interfascicular planes among the actin-IF smooth muscle cells of the bladder detrusor, in such a way to form an intercellular communication network cross talking with the PGP9.5-IF intrinsic nerves. Their location and elongated fashion with branching processes, as well as their contact with nerve fibres, suggest a functional role in the transduction of neuronal inputs and regulation of smooth muscle activity between bladder and urethra during urine storage and voiding. A spontaneous activity was demonstrated to arise in cells located on the boundary of smooth muscle bundles in guinea pig bladder [3]. Contraction waves occurring almost simultaneously along the boundary of smooth muscle bundles are likely to propagate through gap junctions, as it was demonstrated by connexin immunohistochemistry in the guinea pig bladder [3]. 

A relationship of close vicinity of ICs to NOS-I-immunoreactive nerve fibres was occasionally detected at the level of dog bladder. Control of ICs onto the inhibitory, NOS-I-utilizing innervation, has been hypothesized in the guinea pig bladder [1]. In this species, ICs having long dendritic processes extending parallel to the smooth muscle fibres were noticed, which were cGMP-immunoreactive after nitric oxide-mediated induction of cGMP. On this basis, these authors hypothesized that in the guinea pig bladder ICs may be the predominant targets of nitric oxide, which was later confirmed by Lagou et al. [26]. A similar hypothesis has been put forward by Hashitani [16], stating that ICs may be targeted by nitrergic nerves and modulate communications between muscle bundles. Thus, an increase in their population may account for pathological excitability of detrusor smooth muscle.

Nonetheless, nitrergic innervation is not abundant in the dog lower urinary tract, as we previously demonstrated [22]. The contingent of nitrergic nerve fibres, which are present in the lower urinary tract of adult dogs, has a likely local origin, from NOS-IR neuronal bodies located in intramural ganglia. The well-known inhibitory action of nitric oxide could be exerted at this level, directly influencing the cholinergic ganglion cells.

## 5. Conclusions

The finding of ICC-like cells in the canine urinary tract is a noteworthy feature and shows a potential clinical significance, worth of further researches to contribute in understanding the pathogenesis of dog urinary dysfunctional conditions. Investigations aimed at assessing implications of interstitial cells in normal function and neurogenic detrusor overactivity have only been performed on the normal and neuropathic human bladder [27, 28], as well as in a rat model of bladder overactivity [29].

The arrangement of ICs in the canine bladder, contacting the muscle cells and the nerve fibres as well, can rise the question whether they can be proposed as a physiologic pacemaker system. According to the major authors who dealt with the argument [7, 21, 30], several basic requirements must be fulfilled before the hypothesis that certain populations of ICs are pacemakers, that is, generators of slow waves. Basically, it is essential to correlate the presence and distribution of ICs with the presence of slow-wave activity, moreover, pacemaker activity should be present in isolated ICs. At present, it is not possible to infer such a physiologic role with certainty, in the canine bladder, even if it has been proposed for other species [3, 4, 18]. Additional data about expression of cyclooxygenase isoforms in the ICs of rabbit bladder have been obtained recently [31], indicating their possible role as important source of prostaglandins that might likely play a role in regulation of spontaneous rhythmic contractions. The hypothesis of prostaglandin-dependent regulation of spontaneous rhythmic contractions might offer opportunities for the application of novel treatments for disorders leading to overactive bladder.

## Figures and Tables

**Table 1:** Primary and secondary antibodies used for immunohistochemistry and immunofluorescence, their sources, and working dilution.

Primary antisera tested, working dilution	Source and code	Secondary antisera, working dilution	Secondary antisera, working dilution
Mouse Monoclonal Anti-Human:		biotinylated goat anti mouse IgG, 1 : 25	Vector Lab. Inc., USA, BA-9200
C-kit oncoprotein (CD117), 1 : 10	Novocastra, UK, NCL-CD117		
Smooth muscle actin (clone 1A4), 1 : 1000	DakoCytomation, Italy, H-7114		

Rabbit Policlonal Anti-:		biotynilated goat anti rabbit IgG, 1 : 100	Dakocytomation, Italy, Z0421
Protein Gene Product 9.5 (PGP9.5, Ubiquitin C-Terminal Hydrolase), 1 : 1000	Chemicon Int. Inc., USA, AB-1761		
Neuronal nitric oxide synthase (NOS1) (R-20), 1 : 200	Santa Cruz Biotechnology, Inc.,USA, Sc-648		
